# Connaissances, attitudes et pratiques de la population de l'aire de santé SAMBWA en rapport avec le traitement de l'onchocercose à l'ivermectine sous directives communautaires

**DOI:** 10.11604/pamj.2014.19.356.5575

**Published:** 2014-12-08

**Authors:** Cilundika Mulenga Philippe, Yogolelo Asani Bienvenu, Malonga Kaj Francoise, Mukomena Sompwe Eric, Mukalay wa Mukalay Abdon, Oscar Numbi Luboya

**Affiliations:** 1 Faculté de Médecine, Département de Santé Publique, Université de Lubumbashi, Lubumbashi, République Démocratique du Congo; 2 Faculté de Médecine, Service d'Ophtalmologie, Université de Lubumbashi, Lubumbashi, République Démocratique du Congo; 3 Division Provinciale de la Santé du Katanga, République Démocratique du Congo

**Keywords:** Onchocercose, aire de santé, ivermectine, onchocerciasis, health area, ivermectin

## Abstract

**Introduction:**

L'onchocercose constitue un problème majeur de santé publique et l'on a noté environ 13 millions de personnes affectées et 26 millions exposées au cours de l'année 2012 en RDC. L'objectif de notre étude est fournir les données sur le niveau de connaissance, d'attitudes et des pratiques de la communauté huit ans après le lancement de la distribution de l'ivermectine sous directives communautaires.

**Méthodes:**

Il s'agit d'une étude descriptive transversale dans la communauté de l'aire de santé SAMBWA de la zone de santé de KAFUBU. La population cible de l’étude est toute personne de la communauté âgée de 15 à 65 ans. Les paramètres étudiés sont: âge, niveau d'instruction, connaissance de la maladie en langue locale et des signes, attitudes devant les personnes atteintes, perception de la maladie, utilisation des pratiques traditionnelles, association entre le niveau d'instruction et la connaissance de savoir si l'on peut suivre un traitement.

**Résultats:**

La moyenne d’âge des répondants était de 38±14 ans et 67,54% des enquêtés étaient de niveau primaire. L’étude a montré que 99,53% des répondants connaissaient le terme onchocercose en langue locale (UBUMFUKU) et un niveau moyen de connaissance en ce qui concerne les lésions de la peau (66,9%) était noté. 1,9% des répondants craignaient les personnes atteintes et 42,2% des répondants percevaient l'onchocercose comme une maladie. Une proportion de 55% qui prenaient les plantes comme médicament. Il y avait association significative entre le niveau d'instruction et la connaissance de suivre un traitement contre l'onchocercose (p: 0,008).

**Conclusion:**

Ces résultats interpellent en ce qui concerne la sensibilisation de la communauté sur l'onchocercose. Les stratégies de lutte contre l'onchocercose devraient prendre en compte ces différentes insuffisances de la communauté pour améliorer le traitement des masses par l'ivermectine tant au niveau du ménage que des coordinations de programme de lutte.

## Introduction

L'onchocercose (cécité des rivières) est causée par Onchocerca volvulus, transmis à l'homme par la piqûre des mouches noires du genre Simulium, et se caractérise par une maladie chronique de la peau, des démangeaisons sévères, et des lésions oculaires, pouvant évoluer vers la cécité complète. Actuellement, parmi les quelque 123 millions de personnes à risque d'infection dans 38 pays d'endémie, au moins 25,7 millions sont infectés, et 1 million sont aveuglés ou ont une déficience visuelle grave [1]. L'onchocercose provoque des désastres socio-économiques et le Nigeria est le pays le plus endémique dans le monde [2]. Une estimation de 600.000 personnes dans le monde sont aveugles à cause de la maladie et environ 1,5 millions de personnes sont gravement déficients visuels [3]. Le dernier Comité OMS d'experts sur l'onchocercose estime que, en 1995, près de 17,7 millions de personnes ont été infectées, environ 270 000 d'entre eux étaient aveugles et 500 000 gravement déficients visuels [4]. La cécité des rivières ou onchocercose provoque une charge de morbidité considérable en Afrique, principalement à travers la peau et les maladies des yeux [5].

D'après APOC, la RDC est un foyer mondial de la pathologie et environ 13 millions de personnes sont affectées et 26 millions s'y trouvent exposées. Celles qui sont réellement atteintes de la cécité, donc aveugles sont estimées 70 mille. On signale que la RDC a observé le taux de piqûre le plus élevé de moucherons noirs au monde, soit 13 mille piqûres/par jour sur le site d'Inga, dans le Bas-Congo [6]. Et la coordination PNLO Katanga Sud comptait en 2012, 548428 personnes à risque [7]. Toutefois, avant de proposer des mesures correctrices, il est de l'obligation des chercheurs, surtout des coordinations de programme de lutte contre l'onchocercose, d'organiser des enquêtes Connaissance, Attitudes et Pratiques pour déterminer la prise de conscience; les attitudes et le comportement de la communauté face à l'onchocercose et au traitement à l'ivermectine sous directives communautaires.

## Méthodes

Nos recherches se sont effectuées dans l'aire de santé SAMBWA de la zone de santé de KAFUBU. Cette ZS est l'une des 8 ZS que compte le District sanitaire du Haut Katanga. Elle est rurale et nous avions retenu le ménage comme unité d'observation. Nous avons considéré 50% comme prévalence de l'onchocercose dans notre province car depuis le lancement du TIDC, le programme n’étudie plus la prévalence de l'onchocercose. La formule [8] suivante était utilisée pour calculer la taille de notre échantillon:

n= taille d’échantillon requise; zα = niveau de confiance à 95% (pour α = 5%, la valeur de z0, 05= 1,96); p = pourcentage d'utilisation dans la population; i = la précision désirée à 5%

La taille sera estimée par la formule:

N= (Z^2^.p.q)/d^2^

Z = 1,96 au seuil de 5%

p= par le fait que la prévalence de l'onchocercose n'est pas connu dans notre milieu, nous prenons 50%

q = 100% - p = 50%; d = précision = 5%

N=((1.96)^2^ X50X50)/5^2^= 9604/25 = 384

Pour éviter les non répondant au cours de notre étude, nous avons pensé prendre 10% de l’échantillon calculé pour éviter un biais d’échantillonnage. 10% de 384= 38. D'où notre échantillon sera de 384+ 38= 422 ménages.

Le dénombrement des ménages nous a donné pour l'ensemble de l'aire de santé 1398 ménages et pour obtenir notre échantillon, nous avons procédé de la manière suivante: nous avons d'abord subdivisé l'aire de santé en 14 strates correspondant aux 14 villages de l'aire de santé SAMBWA de la zone de santé de KAFUBU. Suite à notre pas de sondage (1398/422), nous avons tiré un chiffre au hasard à l'aide de la table de nombres aléatoires entre 1 et 3 et nous avons procédé au tirage systématique de tous les ménages [9]. Les paramètres étudiés sont: âge, niveau d'instruction, connaissance de la maladie en langue locale et des signes, attitudes devant les personnes atteintes, perception de la maladie, utilisation des pratiques traditionnelles, association entre le niveau d'instruction et la connaissance de savoir si l'on peut suivre un traitement. Les données collectées étaient encodées, saisies, traitées et analysées à l'aide du logiciel Epi info version 7 selon. Le logiciel Excel 2013 était utilisé dans la présentation des variables qualitatives et quantitatives sous forme des tableaux et des graphiques et L'association entre les caractères qualitatifs était mesurée par les tests de chi carré.

## Résultats

La moyenne d’âge des répondants était de 38±14 ans avec un minimum de 15 ans et un maximum de 65 ans. L’étude montre que 67,54% (n = 285) des enquêtés étaient de niveau primaire. Concernant la connaissance du nom onchocercose en langue locale, l’étude a montré que 99,53% (n = 420) des répondants connaissaient le terme onchocercose en langue locale (UBUMFUKU) et seulement 0,47% ignorait le nom de la maladie en langue locale (Tableau 1). Une bonne connaissance des certains signes de l'onchocercose tels que la baisse de la vue (92,6%), la présence des nodules (95%) et les démangeaisons (94,3%) et niveau moyen de connaissance en ce qui concerne les lésions de la peau (66,9%) a été notée (Tableau 2). Une proportion de 98,1% des répondants amenait les personnes atteintes au CS et seulement 1,9% des répondants évitait le contact avec ces personnes car il y avait risque de contagion (Tableau 3). Seulement 42,2% des répondants percevaient l'onchocercose comme une maladie et les autres percevaient l'onchocercose comme un mauvais sort (30,3%) ou comme une malédiction de Dieu (27,5%) (Tableau 4). Parmi les répondants utilisant les pratiquent traditionnelles, une proportion de 55% qui prenaient les plantes comme médicament et les autres (45%) utilisaient les racines (Tableau 5). Par rapport au niveau d'instruction, il y a association significative entre le niveau d'instruction et la connaissance de suivre un traitement contre l'onchocercose (Tableau 6).

**Tableau 1:** Proportion des répondants selon la connaissance du nom onchocercose en langue locale

Connaissance du nom onchocercose en langue locale	Effectif	Pourcentage
OUI	420	99,53
NON	2	0,47
Total	422	100,0

**Tableau 2:** Répartition des répondants selon la connaissance des signes de l'onchocercose

Variables	effectifs	pourcentage
	n-(422)	%
**Baisse de la vue**		
Oui	391	92,6
Non	31	7,4
**Lésions de la peau**		
Oui	282	66,9
Non	140	33,1
**Nodules**		
Oui	401	95
Non	21	5
**Démangeaisons**		
Oui	398	94,3
Non	24	5,7

**Tableau 3:** Répartition des répondants selon leur attitude envers les personnes atteintes ou porteurs de l'onchocercose

Attitudes devant les porteurs d'onchocercose	Effectif	Pourcentage
L'amener au CS	414	98,1
L’éviter car risque de contagion	8	1,9
Total	422	100,0

**Tableau 4:** Proportion des répondants selon leur perception de l'onchocercose

Perception de l'onchocercose	effectif	pourcentage
Une maladie comme toute autre	178	42,2
Un mauvais sort	128	30,3
Une malédiction de Dieu	116	27,5
Total	422	100

**Tableau 5:** Répartition des répondants selon les pratiques traditionnelles utilisées

Pratiques traditionnelles utilisées	Effectif	Pourcentage
les plantes	49	55
les racines	40	45
Total	89	100,0

**Tableau 6:** Association entre le niveau d'instruction et la connaissance de suivre un traitement contre l'onchocercose

Niveau d'instruction		Suivre un traitement contre l'onchocercose		Total	p
		Oui	Non		
Aucune	Effectif	28	2	30	0,008
instruction	%	6,6	0,5	7,1	
Primaire	Effectif	283	2	285	
	%	67,1	0,5	67,6	
Secondaire	Effectif	104	0	104	
	%	24,6	0	24,6	
Supérieur	Effectif	2	1	3	
	%	0,5	0,2	0,7	
Total	Effectif	417	5	422	
	%	98,8	1,2	100,0	

## Discussion

Nos constations à la figure II que la moyenne d’âge des répondants était de 38 ans, (extrême 15 et 65 ans) ceci diffère légèrement de l’étude de Talani qui avait trouvé une moyenne d’âge de 35 ans.(extrême 28 et 65 ans) [10]. Ce léger rapprochement des moyennes s'expliquerait par le fait les individus de cet âge sont souvent les plus actifs dans la société. Une grande proportion de nos répondants (67,5%) avait un niveau d’étude primaire. Ceci s’éloigne des résultats de Banzaoù 58,5% avait un niveau secondaire [11] et de Akilimali à Kinshasa où 55% avait un niveau universitaire [12]. Cet écart pourrait s'expliquer par le cadre d’étude d'Akilimali qui a mené son étude dans la commune de Lemba qui est réputée comme résidence des intellectuels depuis l’époque coloniale. Notre résultat se rapproche de celui de l'enquête menée à Madagascar où la grande majorité des répondants avaient un niveau primaire [13]. Ceci pouvant se justifier par le fait que les deux études ont été menées en milieu rural.

Concernant la connaissance du nom onchocercose en langue locale, l’étude a montré que 99,5% des répondants connaissaient le terme onchocercose en langue locale qu'ils appellent UBUFUNKU; ce résultat se rapproche un peu de celui de Weldegebreal en Ethiopie où95, 9% des répondants connaissaient le terme onchocercose en langue locale (connu localement sous le nom de WARA) [14]. et de celui aussi d'Adeoye au Nigéria où l'onchocercose était bien connu par son nom local ANARUN parmi 91,6% des répondants [2]. Nous pouvons expliquer cette situation par le fait que la sensibilisation a fait connaitre à la communauté la maladie dans tous ses aspects et les individus ont trouvé facilement un nom à la pathologie. Le Tableau 2 révèle une bonne connaissance chez les répondants des certains signes de l'onchocercose tels que la baisse de la vue (92,6%), la présence des nodules (95%) et les démangeaisons (94,3%) et niveau moyen de connaissance en ce qui concerne les lésions de la peau (66,9%). Ces résultats diffèrent de ceux trouvés par Talani en 2004 qui montre une proportion de connaissance chez les répondants de 76,6% pour les démangeaisons considérées comme principal signe de la maladie [10]. et aussi ceux trouvés par Manafa en 2002 où la proportion de connaissance des signes de l'onchocercose chez les répondants était de 64,4% pour les démangeaisons chroniques, de 34% pour les nodules, de 1,4% pour une mauvaise vision et de 3,6% pour les lésions de la peau [15]. Les signes sont connus dans la majorité des répondants un peu partout et ceci peut s'expliquer par le fait des programmes nationaux s'impliquent pour que les communautés connaissent la pathologie.

Concernant l'attitude envers les personnes atteintes, le résultat nous montre seulement 1,9% des répondants évitant le contact avec les personnes car il y avait risque de contagion; ce résultat s’éloigne de celui d'Adeoye qui nous montre 6,2% des répondants qui évitaient tout contact avec des personnes infectées. [2] Et notre résultat se rapproche de celui de Tchounkeu et al qui nous montre que 4,3% est la proportion des répondants qui évitaient les personnes atteintes [16]. 42,2% des répondants percevaient l'onchocercose comme une maladie et les autres percevaient l'onchocercose comme un mauvais sort (30,3%) ou comme une malédiction de Dieu (27,5%). Ce résultat s’éloigne de celui d'Adeoye en 1996 qui montre 74% des répondants aveugles croyaient que des forces externes qui échappaient à leur contrôle et avait causé la cécité due à l'onchocercose [17]. Et de celui aussi de Ndyomugyenyi en 2009 nous montrant une proportion de 71,9% des répondants qui croyaient que l'onchocercose était une maladie grave [18]. Une proportion élevée des répondants n'utilisait pas les pratiques traditionnelles pour traiter l'onchocercose (78,9%) et 29,9% des répondants étaient traités par des pratiques traditionnelles avec comme raisons avancées: par habitude pour 87,5% et par manque de confiance au mectizan pour 12,5%. Les résultats ci-dessous s'approchent de notre résultat en ce qui concerne l'utilisation des pratiques traditionnelles: Adeoye en 2010, nous montre par son enquête que 31% des enquêtés utilisaient des traitements traditionnels [2] et Talani en 2004 qui révèle par son étude que 23,4% utilisaient un traitement traditionne [10].

Concernant les pratiques traditionnelles utilisées, une proportion de 55% prenait les plantes comme médicament et les autres (45%) utilisaient les racines. Ce qui s’éloignent des résultats d'Adeoye qui montre une proportion de 31% pour tous les répondants utilisant comme pratiques traditionnelles herbes et potions [2]. Notre étude a relevé une association significative entre le niveau d'instruction et la connaissance de suivre un traitement contre l'onchocercose (p: 0,015). Ceci rejoint les résultats de Tomen qui montre que le niveau d'instruction influence le comportement des populations (p-valeur = 0.017) [19].

## Conclusion

Ces résultats interpellent en ce qui concerne la sensibilisation de la communauté sur l'onchocercose et le traitement à l'ivermectine sous directives communautaires en milieu rural. Les stratégies de lutte contre l'onchocercose devraient prendre en compte ces différentes insuffisances de la communauté pour améliorer le traitement des masses par l'ivermectine tant au niveau du ménage que des coordinations de programme de lutte contre l'onchocercose.

## References

[1] Anonyme (2013). Progress toward elimination of onchocerciasis in the Americas - 1993-2012. MMWR Morb Mortal Wkly Rep..

[2] Adeoye AO, Ashaye AO, Onakpoya OH (2010). Perception and attitude of people toward onchocerciasis (river blindness) in South Western Nigeria. Middle East Afr J Ophthalmol..

[3] Eyo J, Onyishi G, Ugokwe C (2013). Rapid epidemiological assessment of onchocerciasis in a tropical semi-urban community, enugu state, Nigeria. Iran J Parasitol..

[4] Boatin BA, Richards FO (2006). Control of onchocerciasis. Adv Parasitol..

[5] Coffeng LE (2013). African Programme For Onchocerciasis Control 1995-2015: model- estimated health impact and cost. PLoS Negl Trop Dis..

[6] Oms/Apoc, L Phare/MCN (2012). Lutte contre l’onchocercose: la RDC classée premier réservoir mondial de la cécité.

[7] Pnlo (2012). Rapport des activités 2012.

[8] Ancelle (2006). Statistique épidémiologie.

[9] Mukalay WM, Wilmet DM, Lubumbashi P-Ud (2007). Statistique appliquée à la santé publique.

[10] Talani FM, Kankou JM, Moyen G (2004). La perception, les connaissances et les attitudes pratiques des populations des zones endémiques. Médecine d’Afrique Noire..

[11] Banza R (2008). Perception du risque lié à l'habitat insalubre en milieu urbain(cas de la commune de Kamalonndo), in Santé et développement.

[12] Akilimali P (2008). Déterminant de l'utilisation de la moustiquaire imprégnée d'insecticide en faveur des enfants de moins de 5ans dans la ville de Kinshasa, in Economie de la santé.

[13] PNLP/Antananarive (2011). Enquête sur les IndicateursIndicateurs du Paludismedu Paludisme 2011. Institut National de la Statistique Antananarive.

[14] Weldegebreal F (2014). Assessment of community's knowledge, attitude and practice about onchocerciasis and community directed treatment with Ivermectin in Quara District, north western Ethiopia. Parasit Vectors..

[15] Manafa OU, Isamah AN (2002). Local knowledge and attitudes about onchocerciasis in Oji-River local government area of Enugu State, Nigeria. Epidemiol Infect..

[16] Tchounkeu YF (2012). Changes in stigma and discrimination of onchocerciasis in Africa. Trans R Soc Trop Med Hyg..

[17] Adeoye (1996). Enquête sur la cécité dans les communautés rurales du sud -ouest du Nigeria. Trop Med Int Health.

[18] Ndyomugyenyi RA, Byamungu, Korugyendo R (2009). Perceptions on onchocerciasis and ivermectin treatment in rural communities in Uganda: implications for long-term compliance. Int Health.

[19] Tomen HN (2008). La mouhe noire et les comportements des populations: cas du bassin nyong sanaga au cameroun, in gestion.

